# Nutritional Interventions for Enhancing Sleep Quality: The Role of Diet and Key Nutrients in Regulating Sleep Patterns and Disorders

**DOI:** 10.1002/fsn3.71309

**Published:** 2025-12-04

**Authors:** Rony Abou‐Khalil

**Affiliations:** ^1^ Faculty of Arts and Sciences Holy Spirit University of Kaslik Jounieh Lebanon

**Keywords:** chrononutrition, functional foods, nutritional interventions, personalized nutrition, sleep quality

## Abstract

Sleep disorders and poor sleep quality are increasingly recognized as global health concerns, with substantial consequences for mental and physical health. While pharmacological treatments are available, growing evidence suggests that nutritional interventions offer effective, sustainable alternatives for enhancing sleep quality. This review aims to synthesize current evidence on the impact of key nutrients, dietary patterns, bioactive compounds, and gut microbiome modulation on sleep regulation, and to explore emerging personalized nutrition approaches for managing sleep disorders. A comprehensive review of clinical trials, observational studies, and mechanistic research published over the past two decades was conducted. Key focus areas included sleep‐supportive nutrients, dietary patterns (e.g., Mediterranean, ketogenic, plant‐based diets), chrononutrition, gut‐brain axis modulation, functional foods, and personalized nutrition strategies. Evidence supports the role of specific nutrients (e.g., magnesium, tryptophan, omega‐3 fatty acids) and dietary patterns rich in anti‐inflammatory and antioxidant compounds in improving sleep outcomes. Functional foods such as tart cherry juice and kiwifruit demonstrate potential benefits. The gut microbiome emerges as a significant regulator of sleep physiology, suggesting probiotics and prebiotics as novel interventions. Personalized nutrition approaches, incorporating genetic, metabolic, and lifestyle factors, offer promising individualized solutions. Nutritional interventions represent a promising, non‐pharmacological strategy for improving sleep quality and managing sleep disorders. Future research should focus on personalized approaches and large‐scale clinical trials to validate and refine these strategies for clinical practice.

## Graphical abstract

This review highlights the growing evidence linking diet and key nutrients with sleep quality and circadian regulation. Nutrients such as tryptophan, magnesium, and omega‐3 fatty acids, along with dietary patterns like the Mediterranean and plant‐based diets, demonstrate positive associations with sleep duration and efficiency. Integrating Chrononutrition principles and gut–brain interactions, the paper emphasizes personalized, nutrition‐based strategies as promising, non‐pharmacological approaches for improving sleep health and managing sleep disorders.

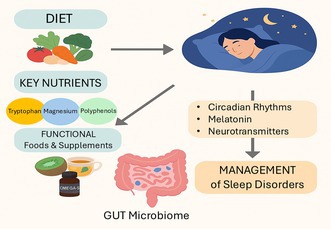

## 1 Introduction to Sleep Physiology and Dietary Influence

Sleep is a fundamental biological process essential for maintaining optimal physical, emotional, and cognitive health. It is characterized by complex neurophysiological changes regulated by intricate interactions between the central nervous system, endocrine signals, immune function, and metabolic processes. Sleep architecture, traditionally divided into rapid eye movement (REM) and non‐REM (NREM) stages, follows an ultradian rhythm and is tightly governed by both homeostatic sleep pressure and circadian rhythms (Circadian rhythm is the body's internal 24‐h biological clock that regulates physiological and behavioral processes such as sleep–wake cycles, hormone secretion, and metabolism in alignment with the light–dark cycle) (Hirshkowitz et al. 2015).

The circadian rhythm, an endogenous, approximately 24‐h cycle, is orchestrated by the suprachiasmatic nucleus (SCN) in the hypothalamus. This internal clock synchronizes with external environmental cues, primarily light, but emerging evidence suggests that food intake patterns also serve as important zeitgebers (time‐givers) (Cedernaes et al. 2019). Dysregulation of circadian rhythms is closely linked to sleep disturbances, including insomnia, delayed sleep phase disorder, and non‐24‐h sleep–wake rhythm disorder.

Neurochemical regulation of sleep involves a range of neurotransmitters and hormones such as gamma‐aminobutyric acid (GABA), serotonin, dopamine, melatonin, orexin, and adenosine (Dijk and Landolt 2019). Notably, the synthesis and function of many of these neurochemicals are influenced by dietary components. For instance, tryptophan, an essential amino acid derived from diet, is a precursor for serotonin and melatonin—key regulators of sleep onset and maintenance (Fernstrom 2012).

Traditionally, interventions for sleep disorders have relied on pharmacological agents such as benzodiazepines, non‐benzodiazepine hypnotics, and melatonin receptor agonists. However, these treatments often come with side effects, risks of dependency, and limited long‐term efficacy (Krystal 2012). Consequently, there is growing interest in non‐pharmacological strategies, including nutritional interventions, to support sleep health naturally and sustainably.

Recent studies have demonstrated that specific nutrients, bioactive compounds, and overall dietary patterns can significantly influence sleep quality, latency, duration, and architecture. Macronutrient composition (ratios of carbohydrates, proteins, and fats) has been shown to impact sleep onset and REM sleep proportions (St‐Onge et al. 2016). For example, diets high in carbohydrates, particularly those with a high glycemic index consumed close to bedtime, may facilitate faster sleep onset, likely due to enhanced tryptophan transport across the blood–brain barrier (Afaghi et al. 2007).

Micronutrients such as magnesium, zinc, and iron are critical for neurotransmitter regulation and have been associated with improvements in sleep quality and reductions in nocturnal awakenings (Zhang et al. 2025; Cherasse and Urade 2017; Gholipour Baradari et al. 2017; Abbasi et al. 2012). Similarly, polyphenols—especially flavonoids found in fruits, vegetables, and teas—exhibit antioxidative and anti‐inflammatory properties that may modulate sleep by influencing GABAergic pathways and reducing neuroinflammation (Bravo 1998).

Beyond individual nutrients, overall dietary patterns also play a crucial role in sleep regulation. The Mediterranean diet, rich in fruits, vegetables, whole grains, lean proteins, and healthy fats, has been correlated with better sleep quality and reduced incidence of insomnia symptoms (St‐Onge et al. 2016). Emerging research into Chrononutrition (Chrononutrition is the study of how the timing, frequency, and composition of food intake interact with the body's circadian system to influence metabolic and sleep–wake regulation), the timing of food intake, highlights that when we eat may be just as important as what we eat, with irregular meal timing potentially disrupting circadian alignment and impairing sleep (Pot 2018).

The gut‐brain axis has recently emerged as another critical interface linking diet to sleep regulation. The gut microbiota produces metabolites such as short‐chain fatty acids and neurotransmitters that can influence central sleep regulation. Dietary fibers, probiotics, and prebiotics have shown potential in modifying the gut microbiome composition in ways that favorably impact sleep quality (Benedict et al. 2016).

Given the increasing prevalence of sleep disturbances and the limitations associated with conventional pharmacotherapies, nutritional interventions represent a promising, accessible, and sustainable approach to improving sleep health. This review will comprehensively explore current evidence on how specific nutrients, dietary patterns, and functional foods can modulate sleep physiology, offering insights into novel, diet‐based strategies for managing sleep disorders. Unless otherwise stated, relationships described in this review are associative and should not be interpreted as evidence of direct causality.

## 2 Methods

### 2.1 Literature Search Strategy

A comprehensive review of the literature was conducted to explore the role of dietary interventions and specific nutrients in regulating sleep patterns and addressing sleep disorders. We systematically searched peer‐reviewed clinical trials, observational studies, and mechanistic research published over the past two decades. The search was performed using three major scientific databases: PubMed, ScienceDirect, and Google Scholar. Search terms included combinations of the following keywords: “sleep quality,” “sleep disorders,” “dietary patterns,” “nutritional interventions,” “functional foods,” “probiotics and sleep,” “tryptophan and melatonin,” “circadian rhythms and diet,” and “gut microbiome and sleep.”

From an initial pool of 1327 articles retrieved, studies were screened based on titles and abstracts. After removing duplicates and applying inclusion and exclusion criteria, a total of 161 key studies were selected for full analysis in this review.

### 2.2 Inclusion Criteria

Studies were included if they met the following criteria:

- Human or animal studies examining the relationship between diet, specific nutrients, dietary patterns, or gut microbiota and sleep parameters.
- Clinical trials (randomized controlled trials [RCTs] or open‐label trials), cohort studies, cross‐sectional studies, case–control studies, or mechanistic experimental studies.
- Reported specific outcomes related to sleep quality, sleep latency, sleep duration, circadian rhythm alignment, or sleep disorders such as insomnia, obstructive sleep apnea, or restless leg syndrome.
- Provided clear methodological descriptions and statistically relevant findings.

### 2.3 Study Selection and Screening Criteria

The selection process involved two independent reviewers who first screened the titles and abstracts for relevance. Studies not focused on diet‐sleep interactions or those lacking primary data (e.g., editorials, narrative reviews without new analysis) were excluded. Full‐text review was performed for articles meeting initial criteria, with discrepancies resolved through discussion or consultation with a third reviewer. The final set of studies was categorized into five main thematic areas corresponding to the review's structure: key nutrients, dietary patterns, gut microbiota interactions, functional foods, and personalized nutrition.

### 2.4 Study Designs and Dietary Focus

Included studies spanned a range of designs:

- *Randomized Controlled Trials* (*RCTs*) evaluating specific food interventions (e.g., tart cherry juice, kiwifruit, omega‐3 supplementation) on sleep outcomes.
- *Observational Cohort Studies* analyzing associations between dietary patterns (e.g., Mediterranean, ketogenic, plant‐based diets) and sleep quality.
- *Cross‐Sectional Analyses* linking nutrient intake (e.g., tryptophan, magnesium) with sleep parameters.
- *Mechanistic Studies* investigating how specific nutrients or gut microbiota influence neurotransmitter production, melatonin synthesis, inflammation, and circadian regulation.

Special focus was given to interventions or exposures involving amino acids, minerals, polyphenols, probiotics, prebiotics, functional foods, and timing of food intake (chrononutrition).

### 2.5 Data Extraction and Primary Outcomes

Data were systematically extracted using a structured template capturing the following elements:

- Study design and sample size
- Population characteristics (e.g., healthy individuals, patients with insomnia, sleep apnea sufferers)
- Type and duration of dietary intervention or exposure
- Specific nutrients, foods, or dietary patterns examined
- Sleep‐related outcomes measured (e.g., sleep quality, latency, efficiency, REM/non‐REM distribution)
- Key findings, effect sizes, and statistical significance
- Proposed biological mechanisms (e.g., neurotransmitter modulation, circadian gene expression, gut‐brain axis communication)

The primary outcomes of interest were:

- Changes in sleep duration, latency, and efficiency
- Improvement or deterioration in subjective sleep quality
- Alterations in circadian rhythm parameters
- Mechanistic insights into diet‐related modulation of sleep architecture

Studies with robust designs (e.g., RCTs, large cohort studies) and strong methodological quality were weighed more heavily in the narrative synthesis, while mechanistic studies were used to support the biological plausibility of observed effects.

This review is a narrative synthesis and does not adhere to PRISMA guidelines. While extensive efforts were made to identify relevant studies, the potential for publication bias and selective inclusion remains. The findings should therefore be interpreted as an integrative overview rather than a systematic meta‐analysis.

While numerous studies indicate associations between specific nutrients and improved sleep outcomes, most evidence remains correlational. Except for well‐controlled randomized trials, causal relationships cannot be conclusively established. Therefore, the terms “is associated with,” “may influence,” or “has been linked to” are used throughout this review to reflect the current level of evidence.

## 3 Key Nutrients and Bioactive Compounds in Sleep Regulation

Emerging evidence underscores the critical role that specific nutrients and bioactive compounds play in modulating sleep architecture and quality. These dietary components influence neurotransmitter synthesis, circadian rhythm regulation, and inflammatory processes—key pathways implicated in sleep physiology. In this section, we will examine amino acids, minerals, vitamins, and polyphenols that have been prominently associated with sleep regulation.

### 3.1 Amino Acids: Tryptophan and Glycine

Tryptophan is an essential amino acid that serves as a precursor for serotonin and subsequently melatonin—two neurotransmitters fundamental to sleep onset and maintenance (Richard et al. 2009). Dietary intake of tryptophan, whether through protein‐rich foods or supplementation, has been associated with shortened sleep latency and improved subjective sleep quality (Hudson et al. 2005). Tryptophan competes with other large neutral amino acids (LNAAs) for transport across the blood–brain barrier; thus, the carbohydrate‐to‐protein ratio in a meal can influence its availability to the brain (Silber and Schmitt 2010).

Glycine, another amino acid, exerts sleep‐promoting effects by acting as an inhibitory neurotransmitter in the central nervous system. Oral glycine supplementation has been shown to improve subjective sleep quality and reduce sleep onset latency, possibly by lowering core body temperature—a physiological signal of sleep initiation (Yamadera et al. 2007).

### 3.2 Minerals: Magnesium, Zinc, and Iron

Magnesium plays a pivotal role in sleep regulation by modulating the activity of N‐methyl‐D‐aspartate (NMDA) receptors and GABAergic systems, both of which are crucial for sleep induction (Abbasi et al. 2012). Magnesium deficiency is linked to increased nocturnal awakenings and reduced slow‐wave sleep, emphasizing its importance for restorative sleep phases.

Zinc, a trace mineral with antioxidant and neuromodulatory properties, has been implicated in the regulation of sleep architecture. A study in infants and preschool‐aged children demonstrated a positive correlation between serum zinc levels and sleep duration (Liu et al. 2024; Giambersio et al. 2023; Sincana et al. 2022; Ji and Liu 2015; Cortese et al. 2009). Zinc may enhance sleep by influencing synaptic plasticity and the modulation of glutamatergic neurotransmission.

Iron is essential for the proper function of dopamine systems, which are critical in regulating sleep–wake cycles. Iron deficiency has been strongly associated with restless leg syndrome (RLS), a disorder characterized by unpleasant sensations and an uncontrollable urge to move the legs during rest (Hening et al. 2009; Allen et al. 2003). Iron supplementation has been shown to alleviate RLS symptoms and improve sleep quality.

### 3.3 Vitamins: Vitamin D and B Vitamins

Vitamin D receptors are expressed in several brain regions involved in sleep regulation, including the hypothalamus. Observational studies have linked low vitamin D status to an increased risk of sleep disorders, particularly short sleep duration and poor sleep efficiency (Faustine et al. 2024; Singh et al. 2024; Abboud 2022; Gao et al. 2018). The mechanistic pathways may involve modulation of inflammatory responses and melatonin synthesis.

The B‐vitamin group, particularly B6 (pyridoxine), B9 (folate), and B12 (cobalamin), plays roles in neurotransmitter metabolism. Vitamin B6 is required for the conversion of tryptophan to serotonin, while folate and B12 are involved in methylation processes that support melatonin synthesis (Li et al. 2023; Puga et al. 2021; Chen et al. 2021; Ma et al. 2019). Deficiencies in these vitamins have been linked to sleep disturbances such as insomnia and fragmented sleep patterns.

### 3.4 Polyphenols: Flavonoids and Other Bioactives

Polyphenols, particularly flavonoids found in fruits, vegetables, tea, and cocoa, have gained attention for their neuroprotective and sleep‐promoting properties. Flavonoids such as apigenin (found in chamomile) and kaempferol have anxiolytic and sedative effects through interactions with GABA_A receptors (Kramer and Johnson 2024; Salehi et al. 2019; Kim et al. 2012).

Other polyphenolic compounds like resveratrol (found in grapes and berries) and epigallocatechin gallate (EGCG) from green tea exhibit anti‐inflammatory and antioxidant effects that may indirectly benefit sleep by reducing systemic inflammation and oxidative stress (Zhang et al. 2024; Wei et al. 2024; Tseilikman et al. 2024). These actions are crucial, as chronic inflammation and elevated oxidative stress are implicated in insomnia and other sleep disturbances.

### 3.5 Emerging Bioactive Compounds: Melatonin and L‐Theanine

While endogenous melatonin secretion from the pineal gland is the primary driver of circadian sleep–wake rhythms, exogenous melatonin (from supplements or foods like tart cherries) has been increasingly utilized to support sleep, especially for individuals with circadian rhythm disorders (Pigeon et al. 2010). Melatonin supplementation has shown efficacy in reducing sleep onset latency, increasing total sleep time, and improving overall sleep quality.

L‐Theanine, an amino acid predominantly found in green tea, has shown promise in promoting relaxation without sedation. Clinical trials have reported that L‐theanine supplementation improves sleep quality, reduces sleep disturbances, and enhances the recovery phase of sleep through modulation of alpha brain wave activity and GABA levels (Lyon et al. 2011).

### 3.6 Mechanistic Insights Into Nutrient–Sleep Interactions

Nutritional modulation of sleep involves multiple biochemical and neurophysiological pathways that converge on circadian regulation, neurotransmitter balance, and hormone synthesis. Amino acids such as tryptophan act as precursors for serotonin and melatonin, both essential for sleep initiation and maintenance. The conversion of tryptophan to serotonin through tryptophan hydroxylase and subsequently to melatonin via arylalkylamine N‐acetyltransferase (AANAT) and hydroxyindole‐O‐methyltransferase (HIOMT) depends on cofactors such as vitamin B6 and magnesium, which facilitate enzymatic activation (Jenkins et al. 2016; Peuhkuri et al. 2012). Micronutrients including zinc and iron influence dopaminergic and GABAergic transmission, modulating sleep–wake transitions and cortical excitability (Cherasse and Urade 2017).

Dietary omega‐3 polyunsaturated fatty acids (PUFAs) alter neuronal membrane fluidity and enhance serotonin receptor signaling, indirectly improving sleep efficiency and mood regulation (Montgomery et al. 2014; Parletta et al. 2013). Magnesium and calcium further stabilize neuronal excitability through regulation of GABA_A receptor activity, promoting inhibitory neurotransmission and reducing nocturnal awakenings (Abbasi et al. 2012; Rondanelli et al. 2011). In parallel, polyphenols and flavonoids such as tart cherry, cocoa, and kiwifruit exert antioxidant and anti‐inflammatory effects that protect neuronal integrity and upregulate melatonin synthesis (Barforoush et al. 2025).

The gut–brain axis also constitutes a pivotal mechanistic interface whereby microbial metabolites—including short‐chain fatty acids, indole derivatives, and secondary bile acids—modulate serotonin production, vagal afferent signaling, and systemic inflammation, thereby influencing sleep architecture (Chen et al. 2025; Anderson et al. 2017). Diets rich in fermentable fibers, prebiotics, and probiotics such as *Lactobacillus rhamnosus GG* and *Bifidobacterium longum* have been shown to enhance GABA synthesis and melatonin secretion through this pathway (Kezer et al. 2025). Collectively, these mechanisms highlight the interdependence between nutrient availability, neurotransmitter synthesis, and circadian hormone regulation in sleep physiology.

Table 1 summarizes the key nutrients and bioactive compounds most frequently investigated in relation to sleep regulation, including their key outcomes, mechanisms, food sources, evidence type, and overall strength of support.

**TABLE 1:** Micronutrients and sleep outcomes.

Nutrient/compound	Key outcomes	Food sources	Mechanism of action	Study type and population	Sample size (range)	Effect direction	Evidence strength	References
Tryptophan	Increased sleep latency, improved sleep quality	Turkey, dairy, soybeans, pumpkin seeds, oats	Precursor for serotonin and melatonin synthesis	RCTs (insomnia, healthy adults)	30–100	Positive	Strong	Roth et al. (2021) Jenkins et al. (2016)
Magnesium	Improved sleep time and efficiency; reduced cortisol	Leafy greens, almonds, avocado, whole grains	Enhances GABA_A receptor activity; reduces cortisol	RCTs (elderly, insomnia)	40–60	Positive	Strong	Abbasi et al. (2012) Rondanelli et al. (2011)
Zinc	Improved sleep onset latency and quality when combined with melatonin and magnesium	Shellfish, pumpkin seeds, nuts, eggs	Cofactor in melatonin synthesis; neurotransmitter modulation	RCTs and small clinical trials	30–100	Positive	Moderate	Rondanelli et al. (2011) Cherasse and Urade (2017) Jazinaki et al. (2024)
Vitamin D	Deficiency linked to shorter sleep duration; causality unclear	Fortified dairy, eggs, oily fish	Regulates tryptophan hydroxylase and melatonin synthesis	Cross‐sectional and cohort studies	1000–3000	Mixed/Positive	Moderate	Gao et al. (2018) McCarty et al. (2012)
Iron	Supplementation reduced restless leg symptoms, improved sleep	Red meat, lentils, spinach, fortified cereals	Cofactor in dopamine synthesis; linked to restless leg syndrome	Observational and supplementation studies	100–250	Positive (specific)	Moderate	McWilliams et al. (2024) Leung et al. (2020)
Calcium	Low dietary calcium associated with poorer sleep quality	Milk, cheese, yogurt, leafy greens	Regulates melatonin synthesis and neuronal excitability	Cross‐sectional (adults)	1000+	Positive	Moderate	Peuhkuri et al. (2012)
Selenium	Higher selenium intake correlated with better sleep quality	Brazil nuts, fish, whole grains	Antioxidant defense; reduces oxidative stress	Observational (NHANES data)	4000+	Positive	Moderate	Grandner et al. (2013)
Potassium	Improved sleep consolidation in older adults	Bananas, potatoes, beans, yogurt	Supports muscle relaxation and circadian rhythm	Small intervention (older adults)	18	Positive (limited)	Weak/Emerging	Drennan et al. (1991) Okamoto et al. (2025)
B Vitamins (B6, B12)	B6 supports melatonin synthesis; B12 may improve circadian rhythm	Whole grains, eggs, meat, legumes	Cofactors in tryptophan → serotonin → melatonin conversion	Observational/small trials	50–300	Positive (B6)	Moderate	Bouloukaki et al. (2023) Peters et al. (2015)
Omega‐3 Fatty Acids	Improved sleep quality, longer sleep duration	Fatty fish (salmon, sardines), flaxseed, chia	Enhances serotonin release; reduces inflammation	RCTs (children and adults)	200–400	Positive	Strong	Montgomery et al. (2014) Parletta et al. (2013)
Polyphenols/Flavonoids	Improved sleep parameters, reduced oxidative stress	Tart cherry, cocoa, berries, kiwifruit, green tea	Antioxidant and anti‐inflammatory; supports melatonin synthesis	Preclinical and clinical studies	50–200	Positive	Moderate	Pérez‐Jiménez et al. (2023) Hibi (2023) Besedovsky et al. (2019) Godos et al. (2025) Calcaterra et al. (2023)
Melatonin (Exogenous/Dietary)	Reduced sleep onset latency, enhanced total sleep time	Tart cherry, walnuts, grapes, tomatoes	Synchronizes circadian rhythms; improves sleep onset	RCTs, meta‐analyses	100–500	Positive	Strong	Ferracioli‐Oda et al. (2013)

**FIGURE 1:**
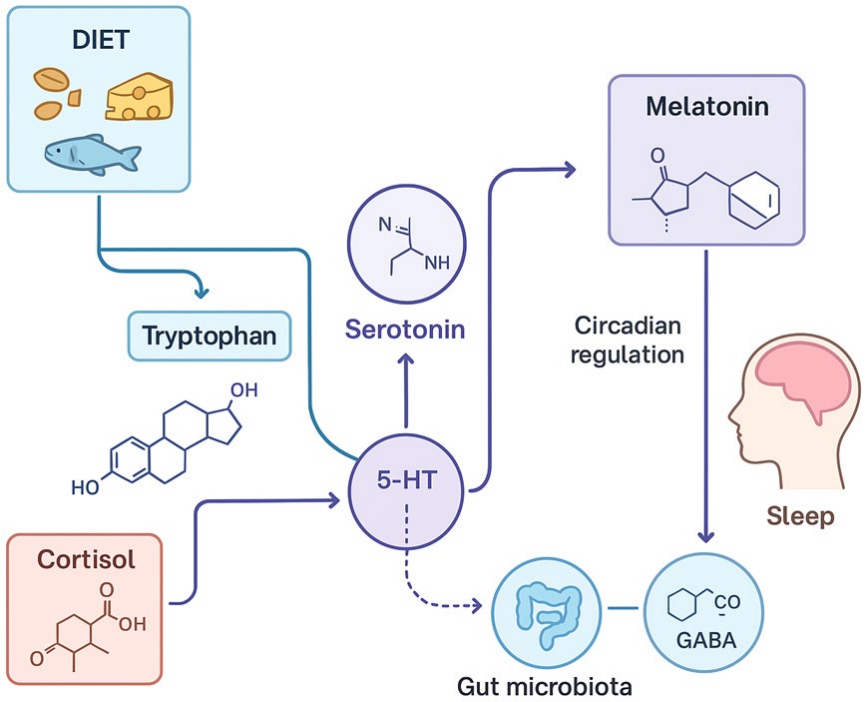
Nutrient–Neurotransmitter Pathways Linking Diet and Sleep Regulation (Kezer et al. 2025; Chen et al. 2025; Barforoush et al. 2025; Anderson et al. 2017; Jenkins et al. 2016; Montgomery et al. 2014; Parletta et al. 2013; Abbasi et al. 2012; Rondanelli et al. 2011). This diagram illustrates the biochemical mechanisms through which dietary nutrients influence sleep physiology. Tryptophan, derived from protein‐rich foods such as fish, cheese, and legumes, is converted into serotonin (5‐HT) and subsequently into melatonin, the hormone responsible for circadian rhythm synchronization and sleep onset. Magnesium, zinc, and other cofactors modulate this pathway by influencing neurotransmitter activity, while gut microbiota contributes via GABA and serotonin signaling. Cortisol interacts antagonistically, linking stress metabolism with sleep disruption.

The biochemical interactions between dietary nutrients, neurotransmitter synthesis, and hormonal regulation are illustrated in Figure 1, highlighting the tryptophan–serotonin–melatonin pathway and the modulatory roles of cortisol and gut microbiota in sleep regulation.

## 4 Dietary Patterns and Their Impact on Sleep Quality

While individual nutrients play a crucial role in sleep regulation, growing research emphasizes the importance of overall dietary patterns in influencing sleep quality and circadian rhythms. Dietary patterns represent habitual combinations of foods and beverages, reflecting synergistic and cumulative effects that may better predict health outcomes compared to isolated nutrients (Hu 2002). In this section, we examine how various dietary models, including the Mediterranean, ketogenic, and plant‐based diets, affect sleep quality and underlying physiological mechanisms.

### 4.1 Mediterranean Diet

The Mediterranean diet (MD), characterized by high consumption of fruits, vegetables, legumes, whole grains, nuts, olive oil, moderate fish and poultry intake, and limited red meat and sugar consumption, has been consistently associated with better sleep outcomes (St‐Onge et al. 2016). Studies suggest that adherence to the MD improves sleep duration, efficiency, and subjective sleep quality, possibly due to its anti‐inflammatory and antioxidative properties (Fallah et al. 2024; Godos et al. 2024; Godos et al. 2019).

Rich in polyphenols, omega‐3 fatty acids, and complex carbohydrates, the MD supports melatonin synthesis, reduces oxidative stress, and enhances serotoninergic neurotransmission—all critical pathways for sleep regulation (Pérez‐Jiménez et al. 2023; Wang et al. 2023; Williamson 2017). Moreover, magnesium‐rich foods, abundant in the MD, may contribute to sleep promotion through their role in NMDA and GABA receptor modulation.

A large cross‐sectional study by Li Rang et al. (2024) reported that higher adherence to the MD correlated with reduced risk of insomnia symptoms in older adults. The complex carbohydrates prevalent in the MD, by increasing the availability of tryptophan to the brain, may facilitate sleep onset and continuity (Afaghi et al. 2007).

### 4.2 Ketogenic Diet

The ketogenic diet (KD), a high‐fat, very low‐carbohydrate diet, has gained popularity for its therapeutic potential in epilepsy, obesity, and metabolic disorders. Recent findings suggest that the KD may also influence sleep architecture. Studies indicate that ketogenic states can increase the proportion of slow‐wave sleep (SWS) and decrease REM sleep, possibly due to alterations in adenosine signaling and energy metabolism (Oleszczuk et al. 2024; Borowicz‐Reutt et al. 2024; Ünalp et al. 2021).

A clinical trial by Hallböök et al. (2007) in children with epilepsy showed that KD improved sleep quality by reducing nocturnal awakenings and enhancing sleep efficiency. Furthermore, ketone bodies themselves may have neuroprotective and anti‐inflammatory effects that stabilize neuronal networks involved in sleep regulation.

However, the KD's impact on sleep can be complex. Initial phases of KD, often associated with keto‐adaptation symptoms (“keto flu”), may temporarily disrupt sleep, suggesting that the duration of adherence and individual metabolic responses are critical factors (Sanderlin et al. 2022; Gangitano et al. 2021; Barrea et al. 2021).

### 4.3 Plant‐Based and Vegetarian Diets

Plant‐based diets, including vegetarian and vegan diets, emphasize the consumption of plant‐derived foods such as fruits, vegetables, legumes, whole grains, nuts, and seeds, with minimal or no animal products. Epidemiological data suggest that plant‐based eaters often report better sleep quality compared to omnivores (Oussalah et al. 2020; Segovia‐Siapco and Sabaté 2019).

The high fiber content of plant‐based diets positively influences gut microbiota composition, enhancing gut‐brain axis signaling, which may contribute to improved sleep (Sejbuk et al. 2024; Lin et al. 2024). Additionally, the anti‐inflammatory profile of plant‐based diets, rich in phytonutrients and antioxidants, may reduce systemic inflammation—a factor linked to poor sleep outcomes (Dashti Hassan et al. 2015; Grandner et al. 2013).

However, careful dietary planning is necessary to prevent potential deficiencies in nutrients like vitamin B12, iron, and omega‐3 fatty acids, all of which are important for optimal sleep regulation. Studies have suggested that lower levels of these micronutrients may impair sleep quality (Gao et al. 2018; St‐Onge et al. 2016).

### 4.4 Chrononutrition: Timing Matters

Emerging research in chrononutrition highlights that not only what we eat but also when we eat has significant effects on sleep health. Irregular meal timing, late‐night eating, and skipping breakfast have been associated with circadian misalignment, delayed sleep onset, and reduced sleep quality (Pot 2018).

Late‐night high‐fat or high‐energy meals can impair sleep initiation and depth by disrupting circadian hormonal rhythms, including melatonin secretion (Scoditti et al. 2022, Bakırhan et al. 2022). Conversely, consuming high‐glycemic index foods approximately 4 h before bedtime may reduce sleep onset latency, suggesting that strategic timing of meals can enhance sleep (Afaghi et al. 2007).

Time‐restricted eating (TRE), a form of intermittent fasting that aligns food intake within a restricted time window during the day, has shown promise in improving sleep efficiency and duration by strengthening circadian rhythms (Manoogian and Panda 2017).

### 4.5 Integrative View: Dietary Patterns and Sleep Health

Taken together, evidence supports that diet quality, meal composition, and timing synergistically influence sleep regulation. Diets emphasizing anti‐inflammatory, antioxidant‐rich foods—such as the Mediterranean and plant‐based diets—promote better sleep outcomes. Meanwhile, attention to meal timing and chrononutritional principles may further optimize sleep quality by reinforcing circadian homeostasis.

**FIGURE 2:**
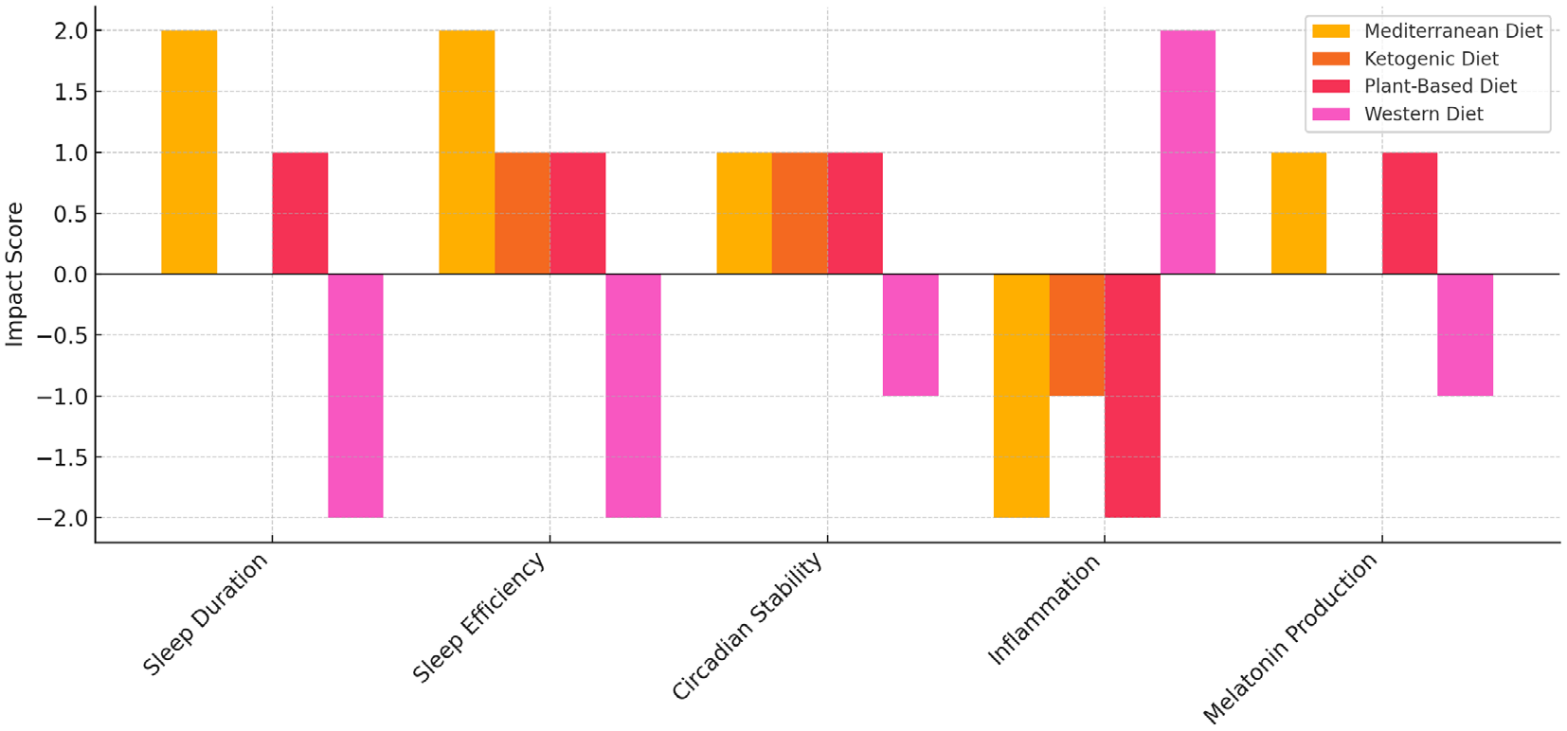
Dietary Patterns and Their Effects on Sleep Outcomes (Pasca et al. 2024; Fallah et al. 2024; Godos et al. 2024; Pattnaik et al. 2022; Zuraikat et al. 2020; St‐Onge et al. 2016; Grandner et al. 2013; Peuhkuri et al. 2012; Benedict et al. 2011; Huang et al. 2002). Impact scores represent the summarized direction and strength of each dietary pattern's effect on sleep outcomes, based on evidence from clinical and observational studies. +2 = Strong positive effect; +1 = Moderate positive effect; 0 = Neutral or mixed evidence; −1 = Moderate negative effect; −2 = Strong negative effect. Scores were derived from a qualitative synthesis of published research and reflect overall trends rather than exact effect sizes.

Figure 2 compares how major diets impact sleep parameters. Mediterranean and plant‐based diets are associated with improvements in sleep duration, efficiency, circadian stability, and melatonin production, along with reduced inflammation. The ketogenic diet shows mixed effects, with potential benefits for sleep efficiency but inconsistent outcomes on duration. Conversely, the Western diet is linked to deteriorated sleep quality and increased inflammation, highlighting the critical role of dietary quality in sleep health (Pasca et al. 2024; Fallah et al. 2024; Godos et al. 2024; Pattnaik et al. 2022; Zuraikat et al. 2020; St‐Onge et al. 2016; Grandner et al. 2013; Peuhkuri et al. 2012; Benedict et al. 2011; Huang et al. 2002).

Thus, dietary interventions aimed at improving sleep should not only focus on individual nutrients but also prioritize holistic dietary patterns and temporal eating behaviors, paving the way for more sustainable, lifestyle‐based strategies to manage sleep disorders.

Table 2 summarizes key dietary patterns and their documented effects on sleep quality, including key outcomes, underlying mechanisms, food sources, study types, and overall evidence strength.

**TABLE 2:** Dietary patterns and sleep quality.

dietary pattern	Key outcomes	Food sources	Mechanism of action	Study type and population	Sample size (range)	Effect direction	Evidence strength	References
Mediterranean Diet	Higher adherence associated with longer sleep duration and lower insomnia risk	Olive oil, fish, whole grains, fruits, vegetables, legumes, nuts	Anti‐inflammatory, antioxidant; supports melatonin synthesis and circadian regulation	Cohort, RCTs (adults, older adults)	1000–3500	Positive	Strong	St‐Onge et al. (2016) Bukari and Qanash (2024)
Plant‐Based Diet	Linked to better sleep efficiency and duration; benefits partly mediated via gut microbiota	Fruits, vegetables, legumes, nuts, whole grains	High in fiber, polyphenols, and antioxidants; supports microbiome balance	Cross‐sectional and RCTs	300–1000	Positive	Moderate–Strong	Sutanto et al. (2020) Clemente‐Suárez et al. (2025) Polianovskaia et al. (2024) Tang et al. (2023) Gan et al. (2024)
Ketogenic Diet	Some improvement in sleep efficiency; REM reduction in some cases	High‐fat, low‐carb, moderate protein; eggs, cheese, meat, oils	Alters GABA/glutamate balance; impacts energy metabolism	Small RCTs (obesity, epilepsy)	20–60	Mixed	Weak–Moderate	Pasca et al. (2024) Masi et al. (2022) Tereshko et al. (2024) Choi et al. (2024) Lounici et al. (2025) Nojek et al. (2024) Ahmad et al. (2024)
Western Diet	Associated with poorer sleep quality, shorter duration, higher insomnia prevalence	Processed foods, red meat, sugar, refined grains, saturated fats	Pro‐inflammatory, disrupts glucose regulation, impairs circadian rhythm	Observational (adolescents, adults)	2000–6000	Negative	Strong (Negative)	Grandner et al. (2013) Clemente‐Suárez et al. (2023)
Chrononutrition (Meal Timing)	Earlier meal timing associated with better sleep onset and circadian alignment	Early vs. late meal distribution; restricted evening eating	Synchronizes feeding and circadian cycles; improves melatonin and insulin sensitivity	Experimental and cohort studies	50–200	Positive	Moderate	Manoogian and Panda (2017) Scoditti et al. (2022) Bakırhan et al. (2022)
High‐Glycemic Index (GI) Diets	Linked to increased nighttime awakenings and shorter sleep duration	Refined carbs, white bread, sugary foods	Increases postprandial glucose → reduces tryptophan transport efficiency	Experimental and observational	100–300	Negative	Moderate	Afaghi et al. (2007)
DASH Diet	Associated with better subjective sleep quality	Fruits, vegetables, whole grains, lean protein, low sodium	Reduces oxidative stress, stabilizes blood pressure, supports sleep regulation	Cross‐sectional (adults, women)	400–800	Positive	Moderate	Liang et al. (2020)

## 5 The Gut Microbiome–Sleep Connection

The gut microbiome, comprising trillions of microorganisms residing primarily in the gastrointestinal tract, plays a critical role in regulating host physiology, including immune function, metabolism, and neural signaling (Cryan et al. 2019). Recently, the gut‐brain axis—a bidirectional communication network between the gut and central nervous system—has emerged as a key player in sleep regulation. This section explores how the gut microbiome influences sleep health and how dietary modulation of the microbiota offers promising avenues for managing sleep disorders (Table 3).

**TABLE 3:** The gut microbiome and sleep regulation.

Component	Mechanisms linking to sleep	Reported sleep effects	Key references
Gut Microbiota Diversity	Higher microbial diversity supports healthy immune function, neurotransmitter production (e.g., serotonin, GABA), and circadian rhythm regulation.	Greater sleep efficiency, reduced sleep fragmentation, improved sleep quality	Lin et al. (2024) Wu et al. (2023) Zhe Wang et al. (2022) Anderson et al. (2017)
Short‐Chain Fatty Acids (SCFAs) (e.g., butyrate)	Produced by microbial fermentation of dietary fiber; modulate blood–brain barrier integrity and influence sleep‐promoting brain regions	Enhanced non‐REM sleep, improved overall sleep architecture	Dalile et al. (2019) Sani et al. (2021) Lin et al. (2024) Queiroz Sarha et al. (2022)
Probiotics	Specific strains (e.g., *Lactobacillus*, *Bifidobacterium*) modulate GABA production, reduce inflammation, and influence cortisol levels	Improved sleep quality, reduced sleep latency, decreased stress‐related sleep disturbances	Lee et al. (2021) Marotta et al. (2019) Azad et al. (2018) Sejbuk et al. (2024)
Prebiotics	Non‐digestible fibers that promote growth of beneficial gut bacteria; impact gut‐derived neurotransmitter production and circadian rhythms	Increased restorative sleep stages, enhanced REM sleep resilience after stress	Thompson et al. (2017) Schmidt et al. (2015)
Gut‐Brain Axis (Microbiota‐Immune‐Neuroendocrine Crosstalk)	Microbial metabolites regulate central nervous system (CNS) functions via vagus nerve activation and hormonal pathways (e.g., cortisol, melatonin)	Balanced sleep–wake cycles, improved mood and cognitive function	Sherwin et al. (2016) Smith et al. (2019)
Dysbiosis (Imbalanced Microbiome)	Promotes systemic inflammation, alters serotonin and GABA synthesis, disrupts circadian gene expression	Poor sleep quality, insomnia symptoms, sleep fragmentation	Lin et al. (2024) Wu et al. (2023) Zhe Wang et al. (2022) Ma et al. (2019)

### 5.1 Gut Microbiota and Sleep: Mechanistic Insights

Emerging research suggests that the gut microbiota can influence sleep through several mechanisms, including modulation of the immune system, regulation of metabolic pathways, production of neurotransmitters, and interaction with the circadian system (Lin et al. 2024; Wu et al. 2023; Zhe Wang et al. 2022; Thompson et al. 2017).

One key pathway involves microbial regulation of inflammatory cytokines, such as interleukin‐6 (IL‐6) and tumor necrosis factor‐alpha (TNF‐α), which are known to affect sleep architecture. Chronic low‐grade inflammation, often associated with gut dysbiosis (imbalanced gut microbiota), has been linked to insomnia and fragmented sleep (Irwin 2015). In contrast, a healthy gut microbiota promotes the production of anti‐inflammatory metabolites like short‐chain fatty acids (SCFAs), which have been associated with improved sleep continuity and quality (Anderson et al. 2017).

Additionally, certain gut bacteria synthesize key neurotransmitters, including gamma‐aminobutyric acid (GABA), serotonin, and dopamine, all of which are critical for sleep regulation (Clarke et al. 2013). Approximately 90% of the body's serotonin, a precursor to melatonin, is produced in the gut, emphasizing the microbiome's influence on circadian and sleep–wake cycles (Yano et al. 2015).

### 5.2 Circadian Rhythms and the Microbiome

The gut microbiota itself exhibits circadian rhythmicity, with microbial composition and function fluctuating throughout the day in response to the host's feeding–fasting cycles and sleep patterns (Thaiss et al. 2014). Disruption of the host's circadian rhythm—such as through shift work, jet lag, or irregular eating patterns—can lead to microbial dysbiosis, which in turn exacerbates sleep disturbances (Zarrinpar et al. 2016).

Conversely, maintaining synchronized circadian rhythms through regular sleep schedules and meal timing can promote a healthy microbial community, thereby reinforcing sleep quality and metabolic health. This interplay suggests that interventions targeting the microbiome may be particularly effective in individuals suffering from circadian rhythm sleep disorders.

### 5.3 Probiotics, Prebiotics, and Sleep

Probiotics—live microorganisms that confer health benefits to the host—have shown promise in improving sleep outcomes. Clinical trials indicate that probiotic supplementation with strains like *Lactobacillus* and *Bifidobacterium* species can reduce stress, improve mood, and enhance sleep quality (Huang and Liu 2024; Chu et al. 2023; Okubo et al. 2019; Daisuke et al. 2019; Mohammadi et al. 2016).

A randomized controlled trial by Nishida et al. (2019) demonstrated that a daily intake of *Lactobacillus gasseri* CP2305 significantly improved sleep efficiency and reduced sleep latency in adults with mild sleep disturbances. These effects were attributed to modulation of the hypothalamic–pituitary–adrenal (HPA) axis and reduction in salivary cortisol levels.

Prebiotics—nondigestible food components like inulin, fructooligosaccharides (FOS), and galactooligosaccharides (GOS)—also play a crucial role in shaping the gut microbiota and promoting the growth of beneficial bacteria. Animal studies have shown that prebiotic consumption can increase non‐REM sleep and improve resilience to stress‐induced sleep disruptions, possibly through enhanced SCFA production and altered gut‐brain signaling (Thompson et al. 2017).

### 5.4 Dietary Fibers and Microbial Metabolites

Dietary fibers, found abundantly in fruits, vegetables, legumes, and whole grains, serve as substrates for microbial fermentation, leading to the production of SCFAs such as acetate, propionate, and butyrate. SCFAs have been shown to cross the blood–brain barrier and influence sleep regulation by modulating neuroinflammation and neuroplasticity (Dalile et al. 2019).

Moreover, higher fiber intake has been associated with deeper, more restorative sleep. In a controlled feeding study, St‐Onge et al. (2016) found that greater fiber consumption was linked to more time spent in slow‐wave sleep, whereas higher saturated fat intake was associated with lighter, less restorative sleep.

Thus, increasing dietary fiber intake represents a simple, non‐invasive strategy to modulate the gut microbiome and enhance sleep quality through the endogenous production of beneficial metabolites.

### 5.5 Dysbiosis and Sleep Disorders

Dysbiosis has been implicated in the pathophysiology of various sleep disorders, including insomnia, obstructive sleep apnea (OSA), and restless leg syndrome (RLS). For example, individuals with OSA often exhibit altered gut microbiota composition characterized by reduced microbial diversity and increased abundance of pro‐inflammatory bacteria (Ko et al. 2019).

**FIGURE 3:**
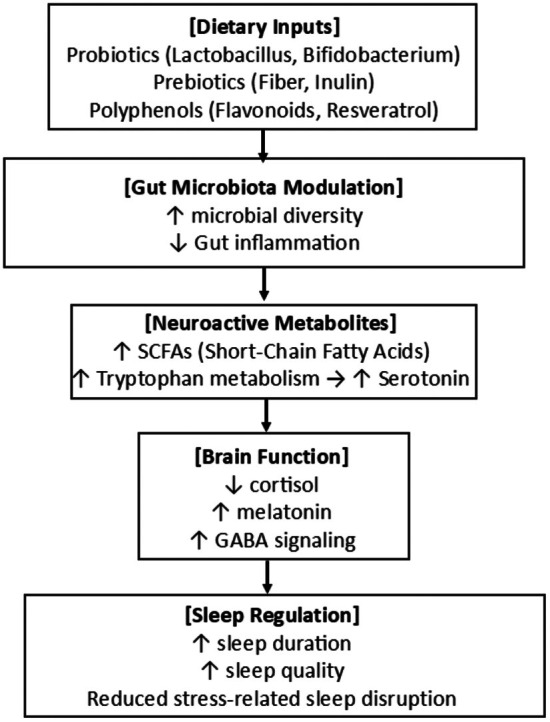
Gut Microbiota–Sleep Axis: Dietary Modulation (Bowers et al. 2022; Smith et al. 2019; Liang et al. 2018; Anderson et al. 2017; Thompson et al. 2017; Aarts et al. 2017; Benedict et al. 2016; Rea et al. 2016; Zheng et al. 2016; Barrett et al. 2012).

Figure 3 shows the emerging role of gut health in sleep regulation. Intake of probiotics, prebiotics, and polyphenol‐rich foods enhances gut microbiota diversity, leading to increased production of neuroactive metabolites such as short‐chain fatty acids and serotonin precursors. These metabolites influence brain function by modulating neurotransmitter levels (e.g., serotonin, GABA) and reducing cortisol and systemic inflammation, collectively contributing to improved sleep quality and duration. This figure highlights emerging pathways linking dietary modulation of the gut microbiome to sleep regulation (Bowers et al. 2022; Smith et al. 2019; Liang et al. 2018; Anderson et al. 2017; Thompson et al. 2017; Aarts et al. 2017; Benedict et al. 2016; Rea et al. 2016; Zheng et al. 2016; Barrett et al. 2012).

Restoring microbial balance through targeted dietary interventions, prebiotics, probiotics, and lifestyle modifications may offer adjunctive therapy for managing sleep disorders, although further clinical trials are needed to establish causality and therapeutic efficacy.

Recent studies have identified specific probiotic strains that exert measurable effects on sleep regulation through modulation of neurotransmitter pathways and stress hormones. *Lactobacillus rhamnosus GG* has been shown to enhance γ‐aminobutyric acid (GABA) receptor expression in the hippocampus and amygdala, promoting anxiolytic and sleep‐promoting effects via vagal signaling (Babaei et al. 2025; Magalhães et al. 2025). Similarly, *Bifidobacterium longum* 1714 and *Lactiplantibacillus plantarum* P8 have demonstrated reductions in cortisol levels and improvements in subjective sleep quality and sleep latency in both clinical and preclinical models (Nishida et al. 2019). These psychobiotic strains also influence serotonin metabolism by increasing tryptophan availability and modulating the kynurenine pathway, thereby enhancing central serotonin synthesis (Xia et al. 2025). Collectively, these findings highlight the potential of targeted probiotic interventions to regulate neurochemical pathways that underlie sleep architecture and circadian alignment.

## 6 Functional Foods and Nutraceuticals for Sleep Enhancement

In recent years, the role of functional foods and nutraceuticals—foods that provide health benefits beyond basic nutrition—has gained attention in the context of sleep health. Several natural products, rich in bioactive compounds, have been shown to modulate sleep quality, latency, and duration through diverse biological mechanisms, including antioxidant activity, anti‐inflammatory effects, and neurotransmitter regulation (Hibi 2023; Bingwei et al. 2022). This section reviews evidence surrounding key functional foods and supplements with potential in sleep enhancement (Table 4).

**TABLE 4:** Functional foods and nutraceuticals for sleep enhancement.

Functional food/nutraceutical	Mechanisms of action	Reported sleep effects	Key references
Tart Cherry Juice	Natural source of melatonin and polyphenols; anti‐inflammatory and antioxidant effects	Improved sleep duration, reduced insomnia severity	Pigeon et al. (2010) Howatson et al. (2012)
Kiwifruit	High in serotonin precursors, antioxidants (vitamin C, E), and folate; supports serotonin and melatonin production	Improved sleep onset, sleep duration, and efficiency	Lin et al. (2011)
Herbal Teas (e.g., Chamomile, Valerian root, Lavender)	Contains apigenin and other bioactives that bind to benzodiazepine receptors; promote GABAergic activity	Shortened sleep latency, improved subjective sleep quality	Zick et al. (2011) Fernández‐San‐Martín et al. (2010)
Omega‐3 Fatty Acids	Modulate serotonin and melatonin synthesis; anti‐inflammatory effects on CNS	Improved sleep quality, especially in children and individuals with mood disorders	Montgomery et al. (2014) Frangou et al. (2006)
Probiotic Supplements	Influence gut‐brain axis by increasing GABA, serotonin; reduce cortisol levels	Improved sleep quality and resilience to stress‐induced sleep disruption	Lee et al. (2021) Marotta et al. (2019) Jangpour et al. (2024) Benton et al. (2007)
Ashwagandha ( *Withania somnifera* )	Contains triethylene glycol and withanolides; acts on GABAergic and serotonergic systems	Enhanced sleep onset and total sleep time; reduced sleep disturbances	Langade et al. (2019)
L‐theanine (Green Tea Amino Acid)	Increases GABA, dopamine, and serotonin; reduces glutamate excitotoxicity	Improved sleep quality, reduced sleep disturbances without sedation	Lyon et al. (2011); Dasdelen et al. (2022) Hidese et al. (2019)

### 6.1 Tart Cherry Juice and Melatonin

Tart cherries ( *Prunus cerasus* ), particularly the Montmorency variety, are a natural source of melatonin, the hormone crucial for regulating the sleep–wake cycle. Tart cherry juice consumption has been linked to improvements in sleep duration and quality due to its melatonin content and antioxidant properties (Pigeon et al. 2010; Howatson et al. 2012).

A randomized, double‐blind, placebo‐controlled trial by Pigeon et al. (2010) found that adults with chronic insomnia who consumed tart cherry juice twice daily experienced modest improvements in sleep continuity. Similarly, Howatson et al. (2012) demonstrated that healthy individuals drinking Montmorency tart cherry concentrate increased sleep time by approximately 34 min and improved sleep efficiency, likely through enhanced endogenous melatonin availability.

### 6.2 Kiwifruit and Serotonin Modulation

Kiwifruit ( *Actinidia deliciosa* ) is rich in antioxidants, vitamins (particularly vitamin C and E), folate, and serotonin precursors, making it a promising food for sleep promotion. Clinical studies have shown that consuming two kiwifruits 1 h before bedtime significantly improves sleep onset, duration, and efficiency in individuals with sleep disturbances (Lin et al. 2011).

The beneficial effects are hypothesized to stem from kiwifruit's high serotonin content, which aids in regulating the sleep cycle by serving as a precursor to melatonin synthesis (Lin et al. 2011). Additionally, the antioxidative capacity of kiwifruit may reduce oxidative stress, which is implicated in the pathophysiology of sleep disorders.

### 6.3 Herbal Teas and Sedative Phytochemicals

Several herbal teas have been traditionally used for their sedative and anxiolytic effects, with chamomile ( *Matricaria chamomilla* ), valerian ( *Valeriana officinalis* ), lavender ( *Lavandula angustifolia* ), and passionflower ( *Passiflora incarnata* ) among the most studied.

Chamomile tea, rich in apigenin—a flavonoid that binds to benzodiazepine receptors in the brain—has been shown to promote sleepiness and reduce nighttime awakenings (Zick et al. 2011). Valerian root contains valerenic acid, which modulates GABAergic activity, thereby inducing sedative effects (Bent et al. 2006). A systematic review by Fernández‐San‐Martín et al. (2010) concluded that valerian preparations significantly improved sleep quality compared to placebo, although variability among studies exists.

Lavender and passionflower teas also demonstrate anxiolytic properties that may contribute to improved sleep through the modulation of central nervous system pathways involved in stress and arousal (Lillehei and Halcon 2014; Koulivand et al. 2013; Nogueira and Vassilieff 2000).

### 6.4 Omega‐3 Fatty Acids and Sleep Regulation

Omega‐3 polyunsaturated fatty acids (PUFAs), especially eicosapentaenoic acid (EPA) and docosahexaenoic acid (DHA), are known for their anti‐inflammatory and neuroprotective properties. Recent evidence suggests that omega‐3 supplementation may also play a role in improving sleep patterns.

Montgomery et al. (2014) conducted a randomized controlled trial in children, showing that higher blood levels of DHA were associated with significantly fewer sleep disturbances. Additionally, supplementation with DHA‐rich fish oil led to longer sleep duration and fewer waking episodes.

The proposed mechanisms include modulation of melatonin secretion, reduction in pro‐inflammatory cytokines, and stabilization of neuronal membranes, all of which are critical for maintaining healthy sleep architecture (Grosso et al. 2014).

### 6.5 Emerging Nutraceuticals: Glycine, L‐Theanine, and CBD

Emerging nutraceuticals are gaining popularity as adjunct therapies for sleep enhancement:

- *Glycine*, a non‐essential amino acid, has been shown to improve subjective sleep quality and reduce fatigue when taken before bedtime. Glycine likely exerts its effects by lowering core body temperature and enhancing vasodilation, key physiological changes for sleep initiation (Bannai et al. 2012).
- *L‐theanine*, a unique amino acid found in green tea ( *Camellia sinensis* ), promotes relaxation without sedation by increasing GABA, dopamine, and serotonin levels. Clinical studies report that L‐theanine supplementation improves sleep quality in individuals with generalized anxiety (Hidese et al. 2017, 2019).
- *Cannabidiol* (*CBD*), a non‐psychoactive compound derived from *Cannabis sativa* , has demonstrated potential for improving sleep through anxiolytic and analgesic effects. Although preliminary evidence supports CBD's role in reducing sleep disturbances, particularly in anxiety‐related insomnia, more robust, long‐term clinical trials are needed (Babson et al. 2017).

## 7 Clinical Evidence and Future Directions in Nutritional Sleep Interventions

As the burden of sleep disorders continues to rise globally, there is a growing recognition of the need for non‐pharmacological strategies to manage sleep disturbances. Nutritional interventions, including targeted supplementation, dietary modifications, and functional foods, have shown promising results in improving sleep outcomes. However, clinical evidence remains heterogeneous, highlighting the need for more rigorous and standardized research. This section synthesizes existing clinical findings and outlines key areas for future exploration.

### 7.1 Current Clinical Evidence on Nutritional Interventions for Sleep

Numerous clinical studies have explored the role of individual nutrients, dietary patterns, and functional foods in modulating sleep parameters. For example, supplementation with melatonin‐rich foods such as tart cherries has consistently demonstrated modest improvements in sleep latency and efficiency in both healthy populations and individuals with insomnia (Pigeon et al. 2010; Howatson et al. 2012).

Similarly, trials investigating magnesium supplementation in older adults and individuals with insomnia have reported enhanced subjective sleep quality and increased sleep duration (Abbasi et al. 2012). Magnesium's regulatory effect on the GABAergic system may partly explain these improvements. Zinc supplementation has also been associated with shorter sleep onset latency and better overall sleep efficiency, particularly when combined with other micronutrients like melatonin and magnesium (Rondanelli et al. 2011).

Dietary interventions, such as adherence to a Mediterranean diet, have shown associations with better sleep quality, longer sleep duration, and lower incidence of insomnia symptoms (St‐Onge et al. 2016). The Mediterranean diet's high content of anti‐inflammatory compounds, fiber, and omega‐3 fatty acids may mediate these positive effects through modulation of systemic inflammation and circadian gene expression.

However, not all studies yield uniformly positive results. Variations in study design, sample size, duration of interventions, and assessment tools (subjective versus objective measures of sleep) contribute to inconsistent findings. Moreover, most studies focus on short‐term outcomes, limiting conclusions about long‐term benefits and sustainability.

### 7.2 Limitations in Current Research

Several limitations impede a comprehensive understanding of how nutritional interventions influence sleep:

- *Heterogeneity of Study Populations*: Many trials include diverse populations varying in age, gender, baseline sleep status, and comorbidities, making it difficult to generalize findings.
- *Lack of Standardized Outcome Measures*: Studies often rely heavily on subjective sleep diaries or questionnaires rather than objective measures such as polysomnography or actigraphy.
- *Short Follow‐up Duration*: Many interventions span only a few weeks, failing to capture the long‐term effects of dietary changes on sleep regulation.
- *Complexity of Nutritional Factors*: Isolating the effect of a single nutrient or food item is challenging due to interactions between various dietary components and lifestyle factors.

Future clinical research must address these limitations to establish stronger causal relationships and inform evidence‐based nutritional guidelines for sleep health.

### 7.3 Emerging Directions in Nutritional Sleep Research

Recognizing the complexity of sleep regulation, future studies are moving toward integrative, multidisciplinary approaches:

- *Personalized Nutrition for Sleep Disorders*: Advances in nutrigenomics and metabolomics are enabling tailored dietary interventions based on individual genetic predispositions, microbiome profiles, and metabolic phenotypes (Sharma and Nelson 2024; BaHammam and Pirzada 2023; Sojeong and Heon‐Jeong 2020; Potter et al. 2016; McEwen and Karatsoreos 2015). For instance, genetic variations in clock genes or melatonin receptor genes may influence responsiveness to certain dietary interventions.
- *Chrononutrition and Timing of Intake*: The timing of food intake profoundly affects circadian rhythms. Emerging studies suggest that time‐restricted feeding, aligning meals with circadian phases, and evening consumption of melatonin‐rich foods can optimize sleep–wake cycles (Pot et al. 2016).
- *Gut‐Brain Axis Modulation*: Given the mounting evidence linking gut microbiota composition with sleep regulation, interventions targeting the microbiome through probiotics, prebiotics, or fiber‐rich diets are gaining interest (Lin et al. 2024; Wu et al. 2023; Zhe Wang et al. 2022)
- *Multi‐Ingredient Approaches*: Instead of focusing solely on single nutrients, future strategies may explore the synergistic effects of combined bioactive compounds (e.g., omega‐3 fatty acids with polyphenols) to amplify benefits on sleep quality.

### 7.4 Need for Large‐Scale, High‐Quality Trials

To advance nutritional sleep medicine, there is an urgent need for:

- Large, multicenter randomized controlled trials (RCTs) with standardized protocols.
- Longer intervention durations to assess the sustainability of sleep improvements.
- Inclusion of diverse populations, particularly vulnerable groups such as shift workers, adolescents, and older adults.
- Use of objective sleep assessment tools (e.g., polysomnography, actigraphy) alongside validated subjective questionnaires.
- Exploration of underlying mechanisms via biomarker analyses, hormonal profiling, and neuroimaging studies.

In conclusion, while current evidence supports the role of diet and specific nutrients in sleep modulation, robust, high‐quality research is needed to translate these findings into clinical practice and public health recommendations.

### 7.5 Methodological Limitations and Confounding Factors in Nutritional Sleep Research

Despite increasing evidence linking nutrition and sleep, several methodological limitations constrain interpretation. First, most data rely on self‐reported dietary intake and sleep quality, which introduces recall and reporting bias. Second, confounding factors such as physical activity, stress, caffeine use, medication, and underlying health conditions often complicate causal inference. Third, interindividual differences in chronotype, age, gender, and genetic polymorphisms (e.g., in CLOCK or PER3 genes) may modify dietary effects on sleep. Finally, short study durations and small sample sizes limit external validity. Future research should standardize assessment tools, integrate objective sleep measures (e.g., actigraphy, polysomnography), and consider these variables to improve reliability.

## 8 Practical Applications and Personalized Nutrition Approaches

Translating scientific findings into actionable strategies is critical for leveraging dietary interventions to improve sleep health. As evidence accumulates regarding the influence of specific nutrients, dietary patterns, and bioactive compounds on sleep, there is an increasing opportunity to apply these insights in clinical practice and individualized health management. This section discusses practical applications and emphasizes the role of personalized nutrition in addressing sleep disorders.

### 8.1 General Dietary Recommendations for Sleep Health

Several broad dietary strategies have emerged from current research that can be recommended to the general population for promoting better sleep:

- *Increase Intake of Sleep‐Supportive Nutrients*: Ensuring adequate consumption of key nutrients such as magnesium, zinc, omega‐3 fatty acids, and tryptophan through a balanced diet can support neurotransmitter synthesis and circadian regulation (Hosseini et al. 2018; Rondanelli et al. 2011).
- *Adopt Anti‐Inflammatory Dietary Patterns*: Emphasizing Mediterranean or plant‐based diets, which are rich in antioxidants, fiber, and anti‐inflammatory compounds, may help improve sleep quality by reducing systemic inflammation and oxidative stress (St‐Onge et al. 2016).
- *Mind the Timing of Meals* (*Chrononutrition*): Aligning eating patterns with circadian rhythms—such as consuming larger meals earlier in the day and avoiding heavy meals close to bedtime—can optimize sleep onset and efficiency (Pot et al. 2016).
- *Limit Sleep‐Disrupting Foods*: Reducing intake of caffeine, alcohol, and high‐glycemic index foods in the evening is important for preventing sleep fragmentation and disturbances (Gardiner et al. 2023; O'Callaghan et al. 2018).

These general recommendations serve as a foundation; however, a “one‐size‐fits‐all” approach may not be adequate for addressing individual variability in sleep needs and responses to dietary interventions.

### 8.2 Personalized Nutrition Approaches for Sleep Disorders

The concept of personalized nutrition—tailoring dietary advice to individual biological, genetic, and lifestyle factors—offers great promise in managing sleep disorders.

- *Genetic Factors*: Polymorphisms in genes involved in circadian regulation (e.g., CLOCK, PER3), melatonin production (e.g., MTNR1B), and nutrient metabolism (e.g., FADS1 for omega‐3 fatty acid synthesis) can influence sleep patterns and responsiveness to dietary interventions (Lopez‐Minguez et al. 2019). Nutrigenetic testing may help identify individuals who would benefit from specific nutrients or chrononutrition strategies.
- *Metabolic Health Status*: Conditions such as obesity, insulin resistance, and metabolic syndrome are closely linked with sleep apnea and other sleep disorders. Personalized interventions that target weight reduction and glycemic control through tailored diets can significantly improve sleep outcomes (Chaput and Tremblay 2012).
- *Gut Microbiota Profiling*: Given the emerging evidence connecting gut microbiome composition with sleep quality, microbiota‐targeted interventions (e.g., personalized probiotic or prebiotic therapy) could be optimized based on an individual's gut microbial profile (Lin et al. 2024; Wu et al. 2023; Zhe Wang et al. 2022).
- *Lifestyle and Behavioral Factors*: Sleep hygiene practices (Sleep hygiene refers to a set of behavioral and environmental practices that promote consistent, high‐quality sleep, including maintaining regular sleep–wake schedules, optimizing the sleep environment, and minimizing stimulants or screen exposure before bedtime), physical activity levels, stress management, and work schedules (e.g., shift work) all interact with dietary patterns. Personalized plans should integrate these behavioral aspects to maximize effectiveness (Chaput et al. 2023; Boege et al. 2021; Scheer et al. 2013).

### 8.3 Personalized Nutrition and Chronotype‐Based Approaches

Emerging research supports the potential for personalized nutrition strategies in sleep health (Konstantinidou and Jamshed 2024). Genetic variations in circadian rhythm–related genes (e.g., CLOCK, PER2, BMAL1) and nutrient metabolism (e.g., FADS1 for omega‐3 synthesis) can influence responsiveness to dietary interventions (Özata Uyar et al. 2024; Franzago et al. 2023). Chronotype differences—morning vs. evening preference—also modify metabolic responses to meal timing, a concept central to Chrononutrition (Campos et al. 2025). Personalized approaches incorporating genetic testing, metabolic profiling, and lifestyle factors could enable precision dietary recommendations for specific sleep disorders such as insomnia, sleep apnea, and delayed sleep phase syndrome.

### 8.4 Future Tools for Personalization: Wearables and Digital Health

Technological advancements, including wearable sleep trackers, digital food diaries, and machine‐learning‐based predictive models, are paving the way for highly personalized and dynamic nutrition‐sleep programs (Leota et al. 2025; Pakwan and Kittiphon 2025; Ka et al. 2025; Wang and Boros 2019; Kelley and Kelley 2017). Wearable devices can monitor real‐time sleep metrics, allowing for immediate feedback and dietary adjustments based on sleep responses. Digital health platforms may also integrate genetic, metabolic, and lifestyle data to generate individualized, adaptive nutrition recommendations.

The integration of personalized nutrition into sleep medicine not only has the potential to enhance individual outcomes but also to contribute to preventive strategies at the population level by identifying at‐risk groups early and intervening proactively.

### 8.5 Barriers and Considerations

While personalized nutrition approaches are promising, several challenges must be addressed:

- *Cost and Accessibility*: Genetic testing and advanced microbiome analysis can be expensive and may not be readily available in all healthcare settings.
- *Data Privacy and Ethical Concerns*: Managing sensitive genetic and health data requires strict privacy protections and ethical oversight.
- *Need for Evidence‐Based Algorithms*: Personalized nutrition recommendations must be based on robust scientific evidence to ensure safety and efficacy.

Addressing these challenges will be essential for the successful integration of personalized dietary interventions into mainstream clinical practice for sleep health management.

### 8.6 Evidence‐Based Dietary Recommendations for Sleep Health

Translating current evidence into actionable dietary strategies is essential for clinicians and individuals seeking to improve sleep quality through nutrition. While dietary responses vary by genetics, lifestyle, and chronotype, several foods and nutrients have demonstrated consistent associations with enhanced sleep duration, efficiency, and circadian stability. Table 5 summarizes practical, evidence‐based dietary recommendations derived from clinical and observational studies, emphasizing whole‐food sources and sustainable approaches to improving sleep health.

**TABLE 5:** Practical dietary recommendations for sleep health.

Nutrient/food category	Food sources	Mechanism of action	Documented sleep effects	Supporting evidence
Tryptophan‐rich proteins	Turkey, dairy, pumpkin seeds, soy products	Serotonin and melatonin precursor	Shorter sleep latency; improved sleep continuity	Bravo (1998); Jenkins et al. (2016)
Magnesium	Leafy greens, almonds, whole grains, avocados	GABA receptor activation; stress reduction	Improved sleep efficiency and total sleep time	Abbasi et al. (2012); Rondanelli et al. (2011)
Zinc	Shellfish, seeds, nuts, eggs	Melatonin synthesis; antioxidant function	Improved subjective sleep quality	Cherasse and Urade (2017)
Omega‐3 Fatty Acids	Fatty fish (salmon, sardines), flaxseed, chia	Enhances serotonin release; reduces inflammation	Longer sleep duration; fewer awakenings	Montgomery et al. (2014) Parletta et al. (2013)
Polyphenol‐rich foods	Tart cherry juice, cocoa, kiwifruit, berries	Antioxidant, anti‐inflammatory, melatonin modulation	Increased total sleep time; improved onset latency	Godos et al. (2025) Calcaterra et al. (2023)
Probiotic foods	Yogurt, kefir, fermented vegetables, kombucha	Gut‐brain modulation of serotonin and GABA	Improved sleep onset and stress resilience	Nishida et al. (2019)
Vitamin D–rich foods	Fortified dairy, fatty fish, eggs	Regulates circadian melatonin synthesis	Mixed but supportive associations with better sleep	Gao et al. (2018); McCarty et al. (2012)
Low‐Glycemic Carbohydrates (evening)	Oats, quinoa, sweet potato	Support tryptophan transport across blood–brain barrier	Faster sleep onset	Afaghi et al. (2007)

## 9 Conclusions

Sleep disorders and poor sleep quality represent significant public health concerns with profound implications for mental, physical, and metabolic health. While pharmacological interventions remain a primary treatment modality, their limitations—including side effects and the risk of dependency—highlight the need for safer, sustainable alternatives. Nutritional strategies, ranging from the intake of specific nutrients and bioactive compounds to the adoption of comprehensive dietary patterns and chrononutrition practices, offer promising avenues for enhancing sleep quality and managing sleep disorders.

Emerging research underscores the influential role of key nutrients such as magnesium, zinc, tryptophan, omega‐3 fatty acids, and polyphenols, as well as the importance of the gut microbiome in sleep regulation. Functional foods and targeted supplementation further expand non‐invasive intervention options. Moreover, the future of sleep health management is likely to be shaped by personalized nutrition approaches, leveraging genetic, metabolic, and lifestyle data to tailor interventions for maximal efficacy.

However, further large‐scale, high‐quality clinical trials are necessary to solidify these findings, refine personalized strategies, and integrate them into mainstream sleep medicine. Nutritional interventions represent a compelling, multifaceted, and patient‐centered approach to improving sleep health, with the potential to significantly impact public health outcomes.

The relationship between nutrition and sleep represents a promising but evolving field. Future studies should prioritize long‐term randomized trials exploring dose–response relationships and mechanistic pathways linking specific nutrients to objectively measured sleep parameters (e.g., EEG‐defined architecture). Integrating dietary interventions with other non‐pharmacological strategies—such as physical activity, light exposure, and stress management—could offer synergistic benefits. Advances in nutrigenomics, metabolomics, and microbiome research will be crucial in developing targeted, personalized nutritional therapies for sleep optimization.

## Author Contributions

Rony Abou‐Khalil solely conceived, designed, researched, and wrote the entire manuscript, including all data synthesis, tables, and figures, and approved the final version for submission.

## Funding

The author has nothing to report.

## Ethics Statement

The author has nothing to report.

## Conflicts of Interest

The author declares no conflicts of interest.

## Data Availability

Data sharing not applicable ‐ the article describes entirely theoretical research.
